# Supplementary material for: A Novel Multi-Gene Combined RT-PCR Assay for Rapid and Sensitive Detection of Maize Dwarf Mosaic Virus
Source: Viruses. 2025 Mar 5;17(3):370. doi: 10.3390/v17030370 (PMC11946660; doi:10.3390/v17030370)
Supplement: Supplementary file 1 [file viruses-17-00370-s001.zip › Figures S1-S7.pdf]

Supplementary Figures

Figure S1: Multiple sequence alignment of the genome (excluding the UTRs) of selected representative MDMV isolates used for primer design. The primer pairs designed in this study are indicated in the alignment. See Table 1 for detailed information on the primers;

Figure S2: RT-PCR amplification of MDMV. Lane M: Maker DNA, Genstar Maker 1000 bp; Lane 1: maize sample; Lane 2–4: positive, negative, and blank controls, respectively;

Figure S3: The results of RT-PCR by multi-gene combined RT-PCR assay. Lane M: Maker, Genstar Maker 1000 bp; Lane 1: 322 bp CP gene amplification product; Lane 2: 343 bp *CP* gene amplification product; Lane 3: 490 bp CI gene amplification products; Lane 4: 640 bp CI gene amplification products; Lane 5: multi-gene combined RT-PCR assay (343 bp and 490 bp); Lane 6: multi-gene combined RT-PCR assay (322 bp and 640 bp); Lane 7–8: negative control of CP gene amplification; Lane 9–10: negative control of CI gene amplification; Lane 11–12: negative control of polygenic combined test;

Figure S4: Optimization of the annealing temperature in multi-gene RT-PCR assay. Lane M: Maker, Genstar Maker 1000 bp; Lane 1–9: annealing temperatures of 45, 47, 49, 51, 53, 55, 57, 59, 61 °C, respectively; Lane 10: negative control;

Figure S5: Optimization of primer dosage in multi-gene combined RT-PCR assay.
Lane M: Maker DNA, Genstar Maker 1000 bp; Lane 1–8: primer dosage combination: G1-G8; Lane 9: negative control;

Figure S6: Products of practical samples by multi-gene combined RT-PCR assay.
(a) 1–15 are maize samples imported from Fuzhou Port, Fujian Province; (b) 1–6 are barley samples imported from Fuzhou Port, and 7–15 are sorghum samples imported from Fuzhou Port; (c) 1–6 are maize samples imported from Xiamen Port in Fujian Province, and 7–15 are sorghum samples imported from Xiamen Port in Fujian Province; (d) 1–15 are sorghum samples imported from Shanghai port. Lane M: Maker, Genstar Maker 100 bp; Lane 1–15: practical application samples; Lane 16,17: negative and blank controls, respectively.

Figure S7: Products of practical samples by RT-PCR. The figure legend is identical to that of Figure **S6**.

Figure S1

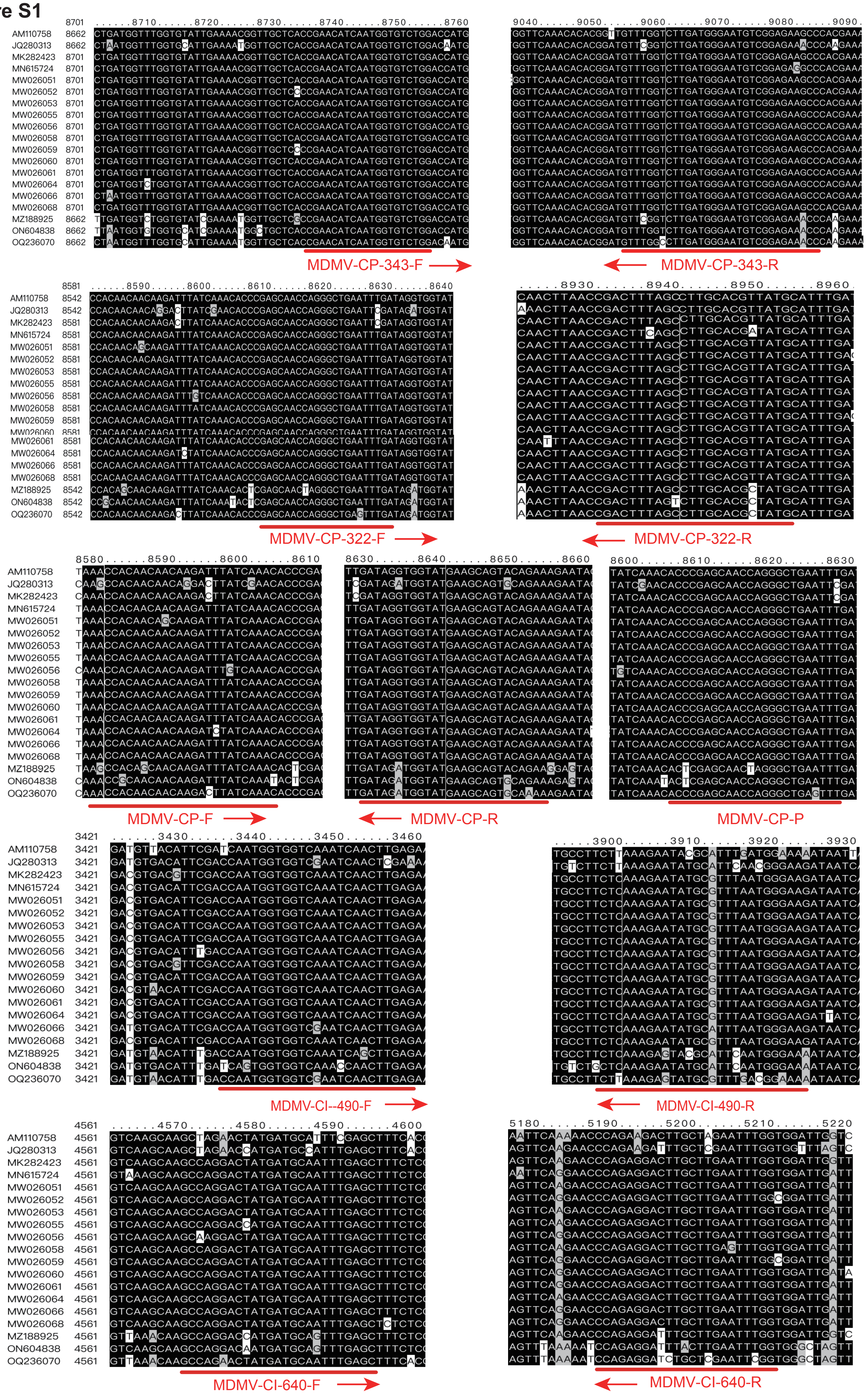
AM110758
JQ280313
MK282423
MN615724
MW026051
MW026052
MW026053
MW026055
MW026056
MW026058
MW026059
MW026060
MW026061
MW026064
MW026066
MW026068
MZ188925
ON604838
OQ236070
MDMV-CP-343-F
MDMV-CP-343-R
MDMV-CP-322-F
MDMV-CP-322-R
MDMV-CP-F
MDMV-CP-R
MDMV-CP-P
MDMV-CI--490-F
MDMV-CI-490-R
MDMV-CI-640-F
MDMV-CI-640-R

Figure S2

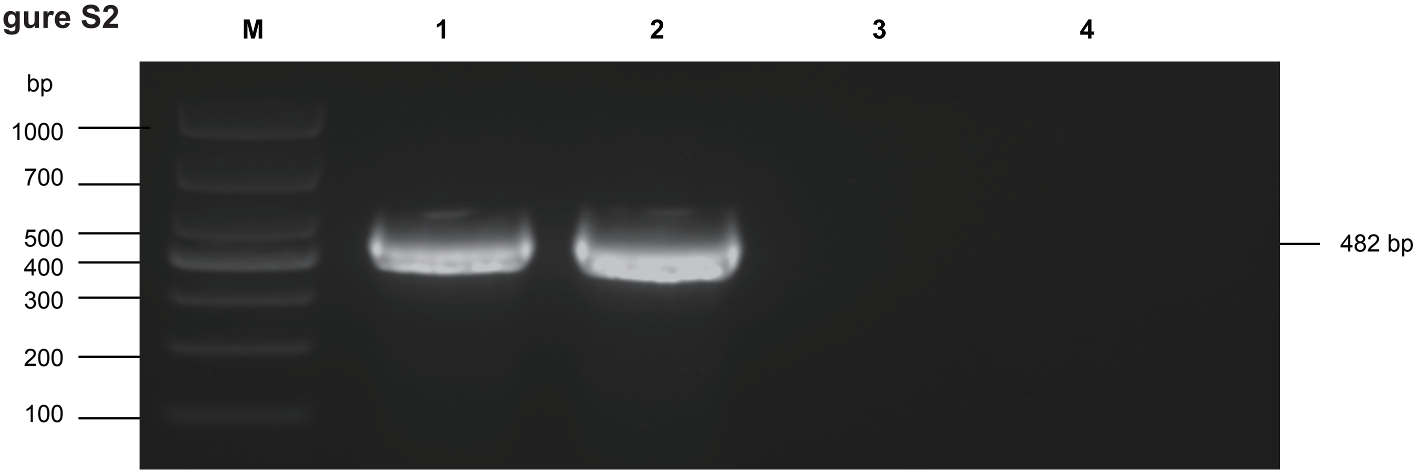

Figure S3

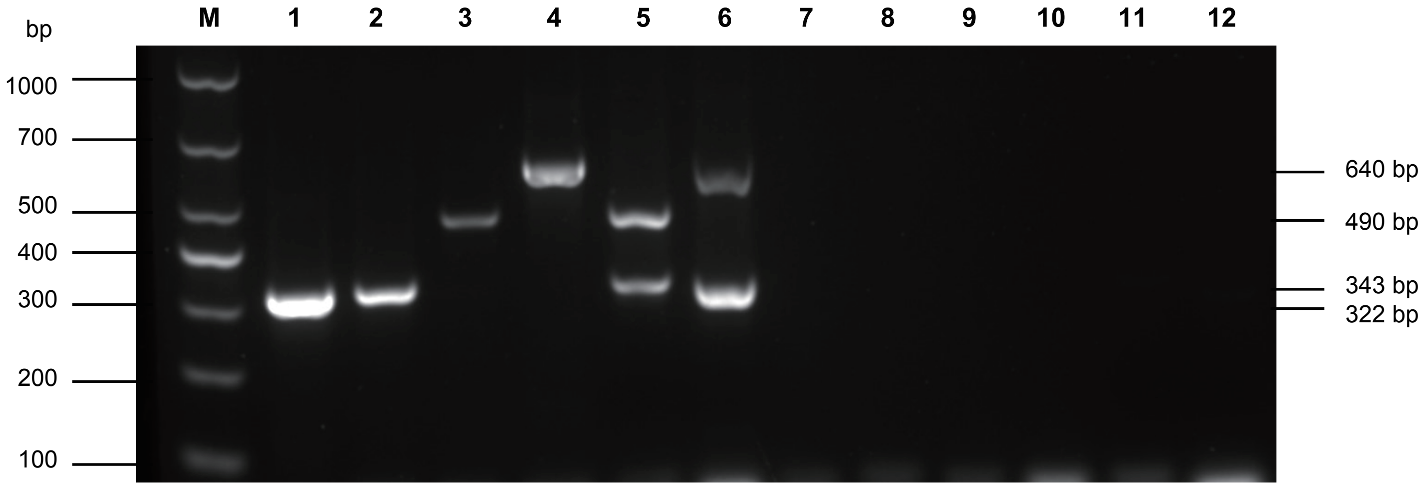

Figure S4

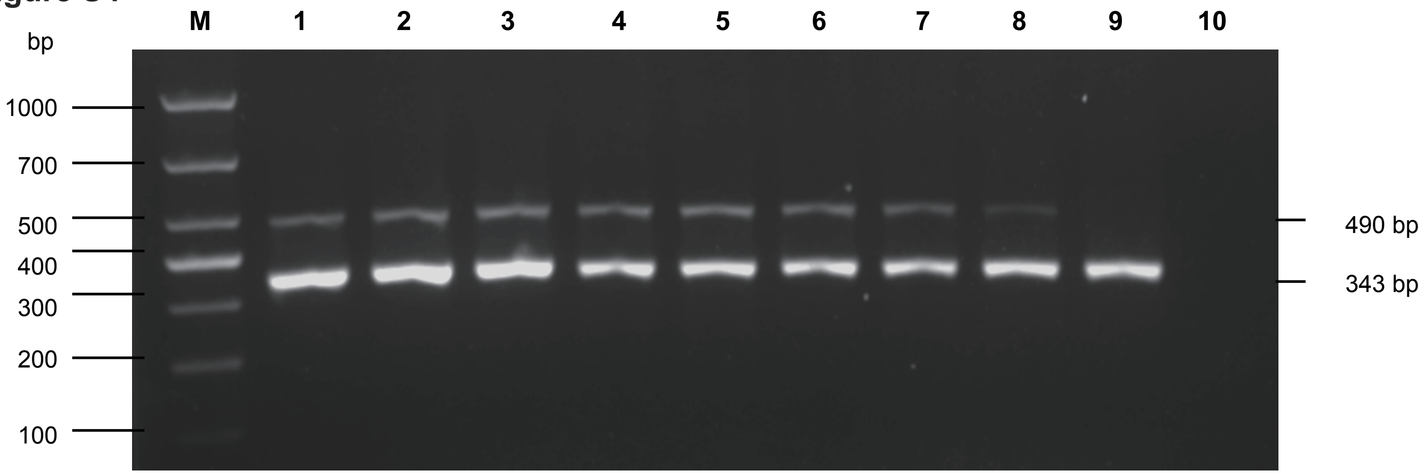

Figure S5

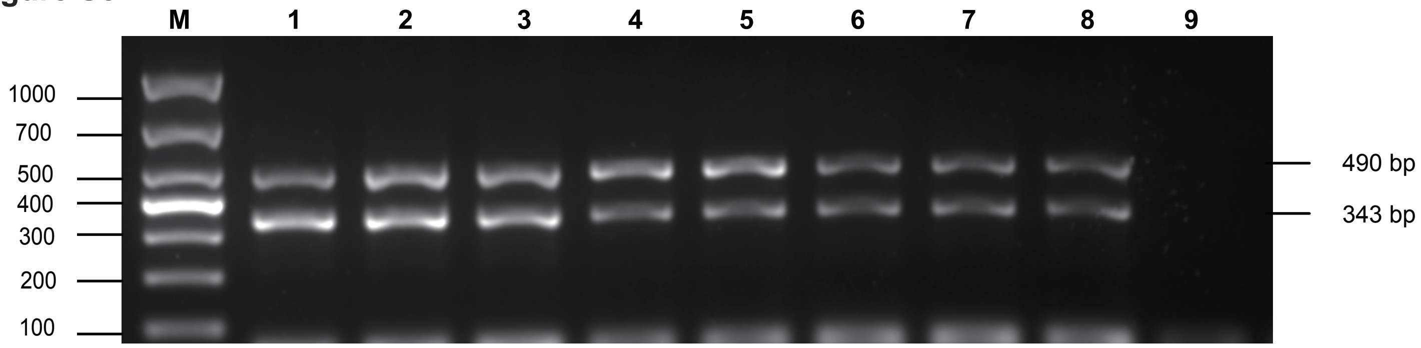

Figure S6

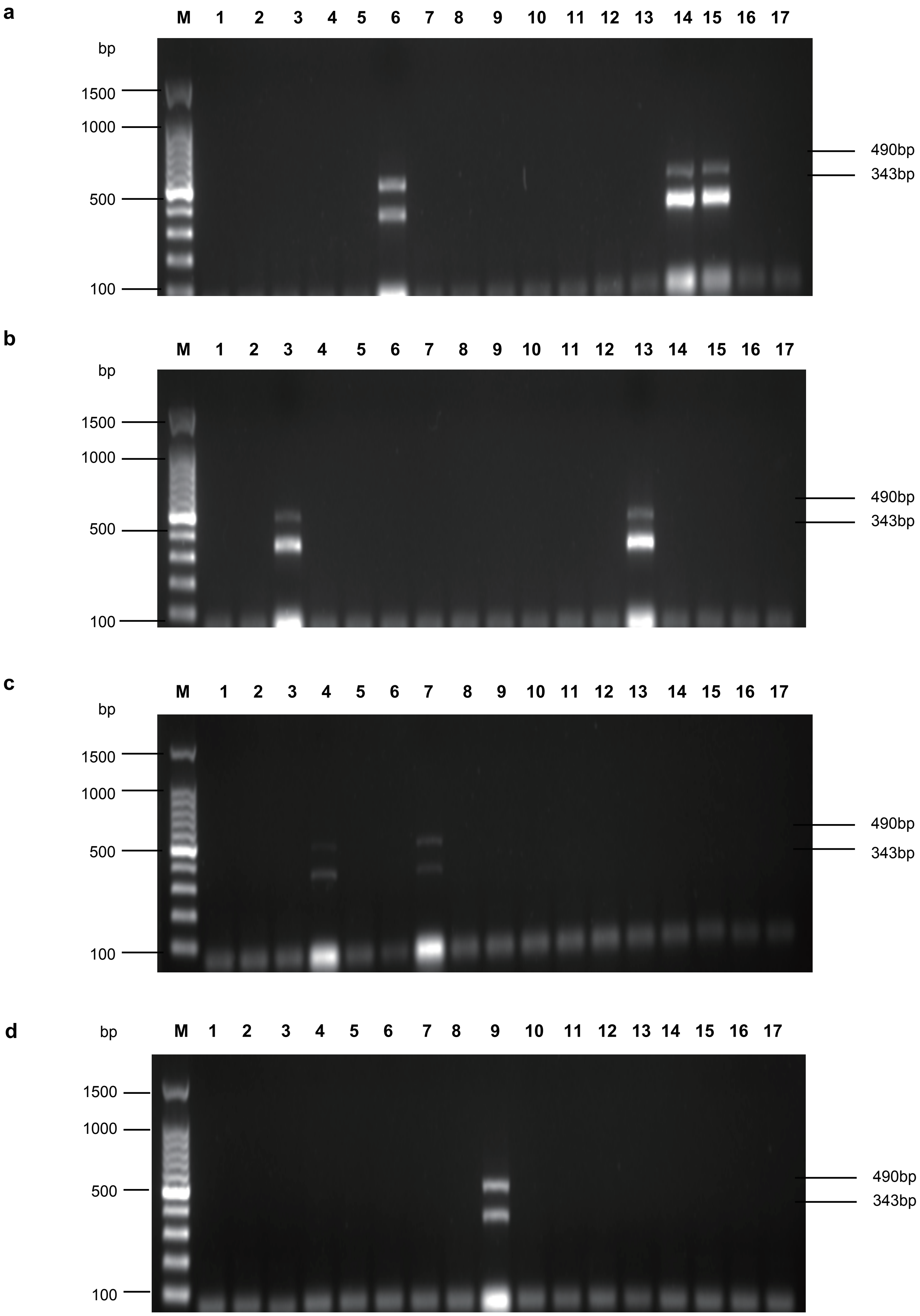
a
M 1 2 3 4 5 6 7 8 9 10 11 12 13 14 15 16 17
bp
1500
1000
500
100
490bp
343bp
b
M 1 2 3 4 5 6 7 8 9 10 11 12 13 14 15 16 17
bp
1500
1000
500
100
490bp
343bp
c
M 1 2 3 4 5 6 7 8 9 10 11 12 13 14 15 16 17
bp
1500
1000
500
100
490bp
343bp
d
bp
M 1 2 3 4 5 6 7 8 9 10 11 12 13 14 15 16 17
1500
1000
500
100
490bp
343bp

Figure S7

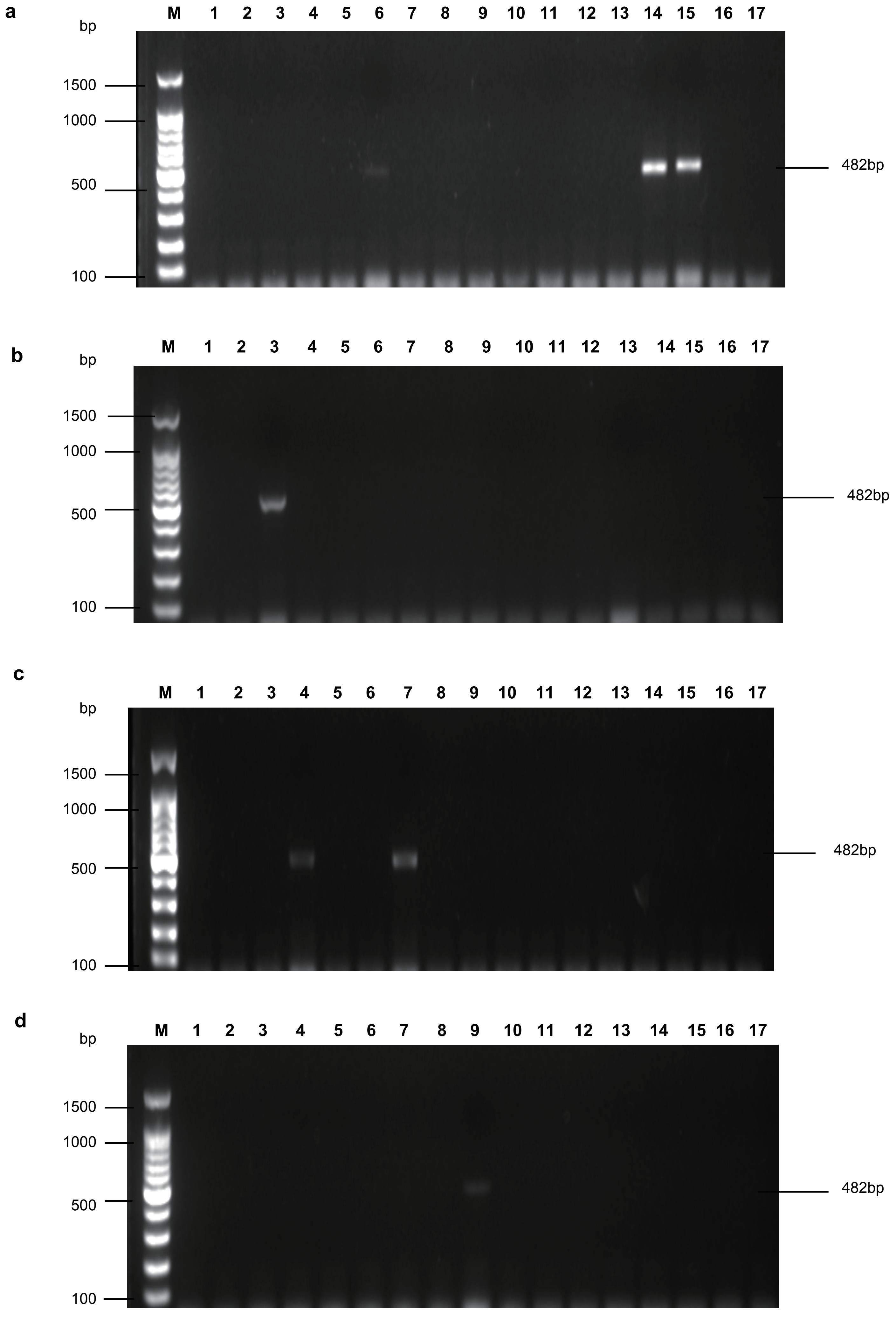
a
M 1 2 3 4 5 6 7 8 9 10 11 12 13 14 15 16 17
bp
1500
1000
500
100
482bp
b
M 1 2 3 4 5 6 7 8 9 10 11 12 13 14 15 16 17
bp
1500
1000
500
100
482bp
c
M 1 2 3 4 5 6 7 8 9 10 11 12 13 14 15 16 17
bp
1500
1000
500
100
482bp
d
M 1 2 3 4 5 6 7 8 9 10 11 12 13 14 15 16 17
bp
1500
1000
500
100
482bp
