# Supplementary material for: A Novel Multi-Gene Combined RT-PCR Assay for Rapid and Sensitive Detection of Maize Dwarf Mosaic Virus
Source: Viruses. 2025 Mar 5;17(3):370. doi: 10.3390/v17030370 (PMC11946660; doi:10.3390/v17030370)
Supplement: Supplementary file 1 [file viruses-17-00370-s001.zip › Tables S1-S4.pdf]

Table S1. List of samples tested in this study.

Number	Serial Number	Port of Entry	Crop	Number	Serial Number	Port of Entry	Crop
1	FZ1	Fuzhou	maize	31	XM1	Xiamen	maize
2	FZ2	Fuzhou	maize	32	XM2	Xiamen	maize
3	FZ3	Fuzhou	maize	33	XM3	Xiamen	maize
4	FZ4	Fuzhou	maize	34	XM4	Xiamen	maize
5	FZ5	Fuzhou	maize	35	XM5	Xiamen	maize
6	FZ6	Fuzhou	maize	36	XM6	Xiamen	maize
7	FZ7	Fuzhou	maize	37	XM7	Xiamen	sorghum
8	FZ8	Fuzhou	maize	38	XM8	Xiamen	sorghum
9	FZ9	Fuzhou	maize	39	XM9	Xiamen	sorghum
10	FZ10	Fuzhou	maize	40	XM10	Xiamen	sorghum
11	FZ11	Fuzhou	maize	41	XM11	Xiamen	sorghum
12	FZ12	Fuzhou	maize	42	XM12	Xiamen	sorghum
13	FZ13	Fuzhou	maize	43	XM13	Xiamen	sorghum
14	FZ14	Fuzhou	maize	44	XM14	Xiamen	sorghum
15	FZ15	Fuzhou	maize	45	XM15	Xiamen	sorghum
16	FZ16	Fuzhou	barley	46	SH1	Shanghai	sorghum
17	FZ17	Fuzhou	barley	47	SH2	Shanghai	sorghum
18	FZ18	Fuzhou	barley	48	SH3	Shanghai	sorghum
19	FZ19	Fuzhou	barley	49	SH4	Shanghai	sorghum
20	FZ20	Fuzhou	barley	50	SH5	Shanghai	sorghum
21	FZ21	Fuzhou	barley	51	SH6	Shanghai	sorghum
22	FZ22	Fuzhou	sorghum	52	SH7	Shanghai	sorghum
23	FZ23	Fuzhou	sorghum	53	SH8	Shanghai	sorghum
24	FZ24	Fuzhou	sorghum	54	SH9	Shanghai	sorghum
25	FZ25	Fuzhou	sorghum	55	SH10	Shanghai	sorghum
26	FZ26	Fuzhou	sorghum	56	SH11	Shanghai	sorghum
27	FZ27	Fuzhou	sorghum	57	SH12	Shanghai	sorghum
28	FZ28	Fuzhou	sorghum	58	SH13	Shanghai	sorghum
29	FZ29	Fuzhou	sorghum	59	SH14	Shanghai	sorghum
30	FZ30	Fuzhou	sorghum	60	SH15	Shanghai	sorghum

Table S2. Different gradient combinations and the primer dosage.

Primer Pair	Different Primer Dosage Gradients(μL)							
	G1	G2	G3	G4	G5	G6	G7	G8
MDMV-CP-343-F/CP-343-R (10 μM)	0.5	0.5	0.5	0.25	0.25	0.25	0.25	0.25
MDMV-CI-490-F/CI-490-R (10 μM)	1.50	2.00	2.50	1.50	1.75	2.00	2.25	2.50

Table S3. Test results of different methods to detect MDMV on imported quarantine samples

Number	Serial Number	Crop	RT-PCR	Multi-Gene RT-PCR Assay	Real-Time Fluorescent Quantitative PCR
1	FZ1	maize	-[1]	-	-
2	FZ2	maize	-	-	-
3	FZ3	maize	-	-	-
4	FZ4	maize	-	-	-
5	FZ5	maize	-	-	-
6	FZ6	maize	+[2]	+	+
7	FZ7	maize	-	-	-
8	FZ8	maize	-	-	-
9	FZ9	maize	-	-	-
10	FZ10	maize	-	-	-
11	FZ11	maize	-	-	-
12	FZ12	maize	-	-	-
13	FZ13	maize	-	-	-
14	FZ14	maize	+	+	+
15	FZ15	maize	+	+	+
16	FZ16	barley	-	-	-
17	FZ17	barley	-	-	-
18	FZ18	barley	-	-	-
19	FZ19	barley	+	+	+
20	FZ20	barley	-	-	-
21	FZ21	barley	-	-	-
22	FZ22	sorghum	-	-	-
23	FZ23	sorghum	+	+	+
24	FZ24	sorghum	-	-	-
25	FZ25	sorghum	-	-	-
26	FZ26	sorghum	-	-	-
27	FZ27	sorghum	-	-	-
28	FZ28	sorghum	-	-	-
29	FZ29	sorghum	-	-	-
30	FZ30	sorghum	-	-	-
31	XM1	maize	-	-	-
32	XM2	maize	-	-	-
33	XM3	maize	-	-	-
34	XM4	maize	+	+	+
35	XM5	maize	-	-	-
36	XM6	maize	-	-	-
37	XM7	sorghum	-	+	+
38	XM8	sorghum	-	-	-
39	XM9	sorghum	-	-	-
40	XM10	sorghum	-	-	-
41	XM11	sorghum	-	-	-
42	XM12	sorghum	-	-	-
43	XM13	sorghum	-	-	-
44	XM14	sorghum	-	-	-

45	XM15	sorghum	-	-	-
46	SH1	sorghum	-	-	-
47	SH2	sorghum	-	-	-
48	SH3	sorghum	-	-	-
49	SH4	sorghum	-	-	-
50	SH5	sorghum	-	-	-
51	SH6	sorghum	-	-	-
52	SH7	sorghum	-	-	-
53	SH8	sorghum	-	-	-
54	SH9	sorghum	+	+	+
55	SH10	sorghum	-	-	-
56	SH11	sorghum	-	-	-
57	SH12	sorghum	-	-	-
58	SH13	sorghum	-	-	-
59	SH14	sorghum	-	-	-
60	SH15	sorghum	-	-	-

[1] - represents a sample that has tested negative; [2]+ represents a sample that tests positive

Table S4. Sequencing results of multi-gene combined detection.

**Number**	**Serial Number**	**Crop**	**Sequencing Result of MDMV-CP-343**	**Sequencing Result of MDMV-CI-490**
1	FZ6	maize	97.12%	94.25%
2	FZ14	maize	95.99%	94.77%
3	FZ15	maize	97.61%	94.21%
4	FZ19	barley	98%	94.77%
5	FZ23	sorghum	97%	93.74%
6	XM3	maize	97.36%	94.7%
7	XM13	sorghum	97.30%	94.18%
8	SH9	sorghum	99%	94.85%
